# Pancreatic Function, Type 2 Diabetes, and Metabolism in Aging

**DOI:** 10.1155/2012/320482

**Published:** 2012-05-17

**Authors:** Zhenwei Gong, Radhika H. Muzumdar

**Affiliations:** ^1^Department of Pediatrics, Divisions of Endocrinology and Geriatrics, Children's Hospital at Montefiore, Albert Einstein College of Medicine, Bronx, NY 10461, USA; ^2^Department of Medicine, Divisions of Endocrinology and Geriatrics, Children's Hospital at Montefiore, Albert Einstein College of Medicine, 1300 Morris Park Avenue, Bronx, NY 10461, USA

## Abstract

Aging is a risk factor for impaired glucose tolerance and diabetes. Of the reported 25.8 million Americans estimated to have diabetes, 26.9% are over the age of 65. In certain ethnic groups, the proportion is even higher; almost 1 in 3 older Hispanics and African Americans and 3 out of 4 Pima Indian elders have diabetes. As per the NHANES III (Third National Health and Nutrition Examination) survey, the percentage of physician-diagnosed diabetes increased from 3.9% in middle-aged adults (40–49 years) to 13.2% in elderly adults (≥75 years). The higher incidence of diabetes is especially alarming considering that diabetes in itself increases the risk for multiple other age-related diseases such as cancer, stroke, cardiovascular diseases, Parkinson's disease, and Alzheimer's disease (AD). In this review, we summarize the current evidence on how aging affects pancreatic **β** cell function, **β** cell mass, insulin secretion and insulin sensitivity. We also review the effects of aging on the relationship between insulin sensitivity and insulin secretion. Understanding the mechanisms that lead to impaired glucose homeostasis and T2D in the elderly will lead to development of novel treatments that will prevent or delay diabetes, substantially improve quality of life and ultimately increase overall life span.

## 1. Introduction

 Aging is an important risk factor for metabolic disorders, including obesity, impaired glucose tolerance, and type 2 diabetes (T2D). Diabetes and its complications remain major causes of morbidity and mortality in the USA [1]. It has been reported that the prevalence of T2D increases with age and peaks at 60–74 [2–4]. Almost one third of the elderly have diabetes and three quarters have diabetes or prediabetes [5]. The higher incidence of diabetes is especially alarming considering that diabetes in itself increases the risk for multiple other age-related diseases such as cardiovascular disease (CVD), atherosclerosis, stroke, Alzheimer disease (AD), Parkinson's disease, nonalcoholic fatty liver disease (NAFLD), and cancer [6]. The pathogenesis of T2D in aging is characterized by two major features: peripheral insulin resistance and impaired insulin secretion from *β* cells [7]. Here, we review how aging predisposes to diabetes and impaired glucose tolerance through effects on insulin secretion and insulin action. 

## 2. Aging and Insulin Secretion

Age-related defects in insulin secretion have been demonstrated in rodents as well as humans. Glucose and amino acid are major stimuli for insulin release from the pancreatic *β* cell. With aging, there is a decrease in insulin secretion following stimulation with glucose as well as amino acid arginine [8]. Decrease in glucose-stimulated insulin secretion (GSIS) *in vivo* has been shown in rodents using the state-of-the-art hyperglycemic clamps [9]. In humans, disorderly insulin release, a decrease in insulin pulse amplitudes, and decreased response to glucose oscillations as well as alterations in insulin clearance have all been demonstrated [10]. When the insulin secretory defects are superimposed over an increased need for insulin as in old age, impaired glucose homeostasis, glucose intolerance, and diabetes can result. We have demonstrated in rodent models that older rodents are unable to increase insulin secretion in proportion to the increased demands imposed by insulin resistance [9], thus contributing to impaired glucose tolerance. Similarly, studies in humans have demonstrated a secretory defect that is consistently observed even after controlling for insulin action. Many factors contribute to the decrease in insulin secretion in aging, including the age-associated loss of Sirt1-mediated GSIS [11], decreased *β*-cell sensitivity to circulating incretins [10], age-associated decrease in mitochondrial function, as well as increased oxidative stress [12]. In this section of the review, we will specifically address age-related decline in various aspects of *β*-cell function and mass that could contribute to the observed defects in insulin secretion.

### 2.1. Aging and Glucose Stimulated Insulin Secretion

**Figure 1:**
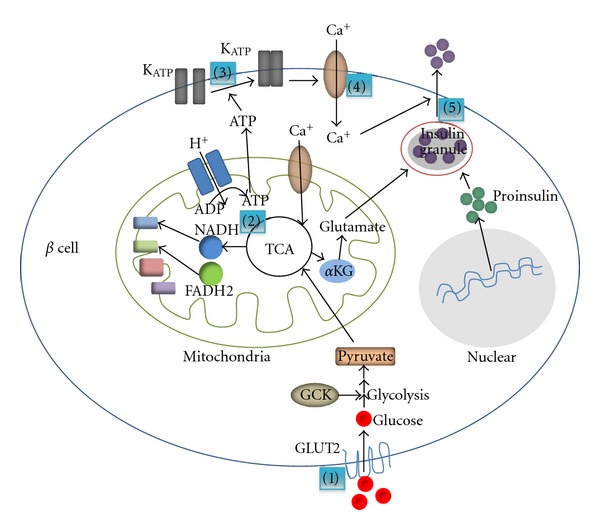
The processes of GSIS. (1) Glucose is transported into the *β* cells through the translocation of the glucose transporters, especially GLUT2; (2) generation of ATP through the oxidation of glucose; (3) elevation the ratio of ATP/ADP induces closure of cell-surface ATP-sensitive K^+^ (K_ATP_) channels, leading to cell membrane depolarization; (4) extracellular Ca^2+^ influx into the *β* cell; (5) a rise in cytosolic Ca^2+^ triggers the exocytosis of insulin granules. K_ATP_: ATP-sensitive K^+^ channels, GLUT2: glucose transporter 2, GCK: phosphorylation by glucokinase, GPDH: glycerophosphate dehydrogenase, TCA cycle: tricarboxylic acid cycle, NADH: reduced form of Nicotinamide adenine dinucleotide, FADH2: reduced forms of flavin adenine dinucleotide, *α*KG: alpha-ketoglutarate, ADP: adenosine diphosphate, ATP: Adenosine-5′-triphosphate.

Insulin, secreted from pancreatic *β* cells, is the major hormone in regulating glucose homeostasis. The secretion of insulin from *β* cells is a complex process involving the integration of multiple stimuli, such as nutrients, hormones, neurotransmitters, and drugs, but the primary stimulus for insulin secretion is circulating glucose. Aging is associated with a marked decline in GSIS in both humans and rodents [13, 14] and the impairment of GSIS is one of the hallmarks of T2D [15]. It is widely accepted that there are five important and “regulatable” steps involved in glucose-induced insulin secretion (as shown in Figure 1): (1) glucose is transported into the *β* cells through the translocation of the glucose transporters (GLUTs), especially GLUT2; (2) generation of ATP through the oxidation of glucose; (3) elevation the ratio of ATP/ADP induces closure of cell-surface ATP-sensitive K^+^ (K_ATP_) channels, leading to cell membrane depolarization; (4) extracellular Ca^2+^ influx into the *β* cell; (5) a rise in cytosolic Ca^2+^ triggering the exocytosis of insulin granules. We will systematically address how aging affects each of these processes.

#### 2.1.1. Aging and Glucose Transporters in *β* Cells

As shown in Figure 1, initiation of the glucose transport is the first step that links glucose metabolism to insulin release in the *β* cell. GLUT2 is the major glucose transporter expressed in pancreatic *β* cells and ensures large bidirectional fluxes of glucose and other dietary sugars, such as fructose and galactose, in and out the cell due to its low affinity and high capacity. Glucose transport is an earlier event in GSIS. Loss of pancreatic *β*-cell GLUT2 expression in humans is associated with hyperglycemia and impaired GSIS [16], and the loss of GSIS directly correlates with decreased expression of the *β* cell GLUT2 in several rodent T2D models [16]. Hou et al. observed that high extracellular glucose concentrations enhance GLUT2 endocytosis, which leads to the insulin secretion in GLUT2 overexpressed *β*-cell line [17]. However, the internalized GLUT2 protein undergoes rapid degradation induced by chronic high-glucose stimulation, which indicates that hyperglycemia directly affects *β* cells function [17]. Moreover, mice lacking GLUT2 in pancreatic *β* cell display an almost complete absence of first phase glucose stimulated insulin secretion [16]. Taken together, these studies suggest that GLUT2 is essential for GSIS and lack of GLUT2 causes hyperglycemia. GLUT2 expression was diminished in very old animals compared with juvenile and adult rhesus monkeys [18], implying the potential connection between age and GLUT2 expression level. Age-associated decrease in expression of GLUT2 has been demonstrated in aged rodent models along with other *β* cell specific genes [19]. The study by Ohneda et al. shows that GLUT2 is underexpressed with increased age; however, neither the magnitude of the underexpression of GLUT2 nor of the reduction in GLUT2 transport function in islets of Goto-Kakizaki (GK) rats is sufficient by itself to explain the profound reduction in GSIS [20]. Compared to rodents, recent evidence demonstrates that human *β* cells express three glucose transporters, GLUT1, 2, and 3 [21]. The higher levels of GLUT1 and GLUT3 may introduce differences in the regulation of glucose sensing in humans versus rodent islets.

#### 2.1.2. Effects of Age on *β*-Cell Glucose Oxidation

After uptake into *β* cells, glucose undergoes oxidation and eventually generates ATP in cytosol and mainly mitochondria via the citric acid cycle also known as the tricarboxylic acid (TCA) cycle, or the Krebs cycle (Figure 1). In the pancreatic *β* cells, glucose oxidation results in the increase of ATP production which is required for insulin secretion. Islets from T2D patients exhibit lower ATP content and blunted GSIS, implicating the mitochondria in the pathogenesis of *β* cell dysfunction [22]. The metabolism of glucose is initiated by its phosphorylation by glucokinase (GCK), a member of a family of evolutionary and structurally related hexokinases. The reaction catalyzed by GCK is the first reaction in glycolysis as well as the first rate-limiting reaction in the metabolism of glucose [23]. GCK mRNA level is markedly decreased in diabetic compared to the normal rats. The gene expression level of GCK was significantly increased with age in healthy rats [24], suggesting a potential mechanism by which *β* cells attempt to overcome age-associated glucose intolerance and insulin resistance. Interestingly, there is evidence to show that glucose oxidation rates are lower in older animals [25]. By using [1-^14^C] and [6-^14^C] glucose-incubated islets isolated from pancreases of 2-month and 12-month-old rats, G. M. Reaven and P. D. Reaven demonstrated that the amount of glucose converted to CO_2_ by islets from 12-month-old rats was only half that of 2-month-old rats [25]. In humans, there is a lower glucose oxidation but higher lipid oxidation rates in elderly than in the young, suggesting that an enhanced Randle cycle may play a major role in producing a reduction in insulin-mediated glucose oxidation [26].

MacDonald has pointed out that pancreatic islets contain 40–70 times the activity of mitochondrial glycerophosphate dehydrogenase (GPDH) compared to other tissues [27], implicating the potential importance of GPDH in islets. Since GPDH plays a crucial role in transporting and reducing equivalents from cytosol to mitochondria, a decrease in its activity leads to an accumulation of cytosolic NADH and consequently, an increase in the cytosolic NAD/NAD+ ratio, a decrease in glycolysis, and a reduction in GSIS. An approximate 50% reduction in the activity of GPDH in islets of 12-month-old compared with 2-month-old rats [28], suggesting a role for GPDH in diminished GSIS in aging. 

#### 2.1.3. Aging and Calcium and Potassium Channels

Increased ATP/ADP ratio from glucose oxidation reduces the whole cell K^+^ permeability, leading to cell membrane depolarization and extracellular Ca^2+^ influx into the *β* cell. These changes are thought to be mediated through the ATP-sensitive K^+^ (K_ATP_) channels and voltage-dependent Ca^2+^ channels (Figure 1). Elevation of cytosolic free Ca^2+^ concentration provides a link with the insulin exocytotic process. Studies from Ammon and colleagues have shown that raising the glucose concentration from 3 to 5.6 and 16.7 mM had no effect on K^+^ efflux from islets of 24-month-old male rats whereas that from 24-month-old female rats were decreased. Also, net uptake of Ca^2+^ was significantly diminished in islets of 24-month-old compared to islets of 3-month-old rats. In the presence of 16.7 mM of glucose, islets of 24-month-old rats exhibited only 60–70% of the insulin release obtained with islets from 3-month-old rats. These data suggest that the decreased insulin secretory response to glucose during old age is due, at least in part, to inadequate inhibition of K^+^ efflux and diminished net uptake of Ca^2+^ [29]. However, it is important to consider that the differences in ion fluxes could relate to age-related deficits in the steps leading up to ion flux such as decreased glucose transport and oxidation.

#### 2.1.4. Effects of Age on Insulin Granule Exocytosis

The final step of insulin secretion is the exocytosis of insulin granules (Figure 1). Insulin is stored in large dense core vesicles (LDCVs), also called insulin granule, and released by exocytosis, a multistage process involving vesicle trafficking, docking, and eventually fusion with the plasma membrane [30]. Calcium constitutes the major stimulus for exocytosis. Ca^2+^ regulates several steps in exocytosis, such as the size of vesicle pools, the fusion event, and the size of the fusion pore, and may act on distinct protein targets [31, 32]. Since the net uptake of Ca^2+^ is decreased with age [29], it is reasonable to speculate that insulin granule exocytosis is also inhibited by age. To date, insulin secretion is known to involve the same soluble N-ethylmaleimide, sensitive factor attachment protein receptor (SNARE) isoforms as those utilized in synaptic vesicle exocytosis and neurotransmitter release, namely, Syntaxin 1, vesicle-associated membrane protein (VAMP) 2, and synaptosomal-associated protein of 25 kDa (SNAP-25) [33–37]. The exocytosis is induced by the pairing of SNARE proteins on the vesicle membrane, termed v-SNARE (such as VAMP2), with cognate proteins on the target membrane, the t-SNAREs (syntaxin and SNAP-25). In addition, other proteins such as munc18/sec1 and munc13 have also been reported to be involved in the regulation of insulin granule exocytosis [38, 39]. Vanguilder and colleagues showed that neurotransmission-regulating proteins such as VAMP2, Syntaxin1, and SNAP-25 decline with age in hippocampal synaptosomes in rats. Altered synaptic protein expression may decrease stimulus-induced neurotransmission and vesicle replenishment during prolonged or intense stimulation, two processes that are necessary for learning and the formation and perseverance of memory [40]. However, it is still unclear whether these proteins also decline with age in pancreatic *β* cells. Further studies are needed to address this question.

### 2.2. Aging and *β*-Cell Mass

**Figure 2:**
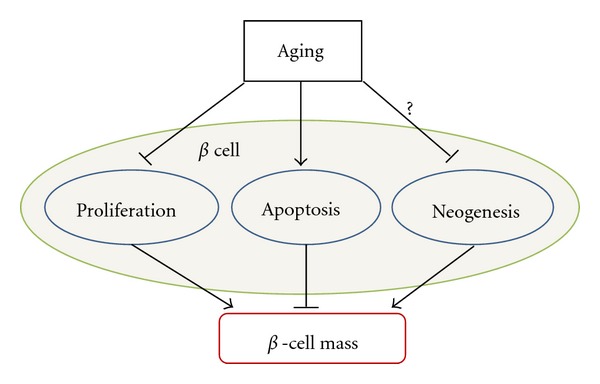
Factors that control *β*-cell mass in aging. *β* cell proliferation, apoptosis, and islet neogenesis are three major factors that control *β*-cell mass. Through differentially regulation of these factors, aging modulates *β*-cell mass, and subsequently insulin secretion.

The proliferation and apoptosis of *β* cells and islet neogenesis are three major factors that tightly regulate *β*-cell mass. As shown in Figure 2, in this section, we will review the effects of aging on these factors.

#### 2.2.1. Apoptosis and Proliferation

Pancreatic *β*-cell mass is mainly controlled by the balance of cell proliferation and apoptosis. It has been shown that age correlates with decreased proliferative activity and enhanced sensitivity to glucose-induced *β*-cell apoptosis [41] (Figure 2). In cultured islets from 2 to 3-month-old rats, increasing glucose from 5.5 to 11.1 mM decreased *β*-cell apoptosis and increased *β*-cell proliferation, which was further augmented when glucose was increased to 33.3 mM. In contrast, in islets from 7 to 8-month-old rats, increasing glucose concentrations from 5.5 to 33.3 mM induced a linear increase in *β*-cell death and a decrease in proliferation. This has also been observed in cultivated human islets where age correlated positively with the sensitivity to glucose-induced *β* cell apoptosis and negatively to baseline proliferation [41]. It is reported that there is a three-to-ten-fold increase in *β*-cell apoptosis in diabetic patients compared to nondiabetic individuals as detected by TUNEL staining [42]. Maedler et al. demonstrated that high glucose induces apoptosis in human pancreatic *β* cells through inducing the expression of Fas and activation of caspase 8 and 3 [43]. Reers et al. report that relative *β*-fcell volume in human pancreatic islets remains constant with aging and *β*-cell replication decreases age dependently, while *β*-cell apoptosis does not change significantly [44]. However, the most recent study performed on 4-month, 14-month and, 24-month-old Wistar rats has shown that there is a significant reduction in *β*-cell proliferation and increase in apoptosis in *β* cells in the islets [45]. The study also showed that age associated decreases in activities of many antioxidant enzymes and suggests that an increase in oxidative stress in the *β* cells contributes to the increased apoptosis [45]. 

Islet amyloid polypeptide (IAPP) or amylin is cosecreted with insulin from *β* cells [46]. Amylin suppresses glucagon secretion and helps to regulate glucose homeostasis. In insulin-resistant states, hypersecretion of insulin results in increased cosecretion of amylin. Careful analyses show that the amylin could aggregate into amyloid plaques that increase *β*-cell apoptosis leading to reduced islet volume and *β*-cell mass [47], and subsequent diabetes [48]. Accumulating evidence implicates toxic IAPP oligomers in the mediation of *β* cell apoptosis in T2D. In support, freshly dissolved human IAPP (hIAPP, but not rodent IAPP) induces apoptosis when added to cells in culture [49]. *β*-cell-specific transgenic overexpression of hIAPP result in hyperglycemia in the mice [50]. Although research has shown that with age there is an increased deposition of amylin in the islets of diabetic individuals but not nondiabetic individuals [2], it is still too early to rule out the potential influence of aging on amylin aggregation.

*β*-cell mass normally grows well into adulthood to provide increased insulin secretion capacity to match the greater insulin requirements of maturity [51]. *β*-cell mass can slowly expand in adult rodents in response to increased insulin requirements or during pregnancy [52]. Several mechanisms have been invoked to explain adult *β*-cell mass expansion, including neogenesis from pancreatic ducts or hematopoietic tissues [53], replication of specialized *β*-cell progenitors, and self-renewal by *β* cell [54, 55]. *β*-cell proliferation and the capacity of *β* cell to regenerate declines with age in mice [56]. Basal *β*-cell proliferation is severely decreased with advanced age. The rate of *β*-cell proliferation gradually declines with aging in rats to a steady state by 7 months of age [13].

Young mice respond to high-fat diet by increasing *β*-cell mass and proliferation and maintaining normal blood glucose levels. Old mice, in contrast, do not display any increases in *β*-cell mass or *β*-cell proliferation in response to high-fat diet and become diabetic. There is a four-fold increase in *β* cell proliferation in young mice after the administration of streptozotocin (STZ), a chemical that is toxic to the *β* cells in mammals and normally is used for inducing insulin secretion deficiency models in rodents, whereas no changes are observed in older mice [56]. Similarly, STZ stimulated *β*-cell replication in young mice but had little effect in old mice [57]. Partial pancreatectomy greatly stimulated *β*-cell proliferation in young mice but failed to increase *β*-cell replication in old mice [57].

p16^Ink4a^ is an effector of senescence [58] and a potent inhibitor of proliferative kinase cyclin-dependent kinase 4 (Cdk4) [59], which is essential for pancreatic *β* cell proliferation in adult mammals [60, 61]. Islets *in vivo* exhibit increased p16^Ink4a^ expression with age in rodents and humans [62]. Another cyclin-dependent kinase, Cdk6, is not expressed in mouse islets but is very effective in driving *β* cell replication in human islets [63]. Ablation of p16 leads to improved *β*-cell function with age [64]. Lack of Cdk4 expression in mice leads to insulin-deficient diabetes [65]. To perform their kinase activity, Cdks bind some kinds of regulatory proteins called cyclins. Without cyclins, Cdks have little kinase activity. Mouse *β* cells express 3 D-cyclins, termed cyclin D1, D2, and D3; D2 is the most abundant one whereas D3 is nearly undetectable [66]. Prenatal islet development occurred normally in cyclin D2^−/−^ or cyclin D1^+/−^ D2^−/−^ mice. However, *β*-cell proliferation, adult mass, and glucose tolerance were decreased in adult cyclin D2^−/−^ mice, causing glucose intolerance that progressed to diabetes by 12 months of age. Although cyclin D1^+/−^ mice never developed diabetes, life-threatening diabetes developed in 3-month-old cyclin D1^−/+^ D2^−/−^ mice as *β*-cell mass decreased after birth. Thus, cyclins D2 and D1 were essential for *β* cell expansion in adult mice [66]. In contrast, in the human *β* cell, cyclin D3 is highly expressed, whereas cyclin D1 and D2 levels are much lower. Overexpression of cyclin D3 in isolated human islets, especially in combination with Cdk6, induced the greatest increase in *β*-cell proliferation when compared with over-expression of other cyclins [63]. Although cell cycle proteins play crucial roles in the proliferation of pancreatic *β* cells, less is known about the effects of aging on the expression or/and activity of cyclins and Cdks; therefore, studies in this area may contribute to a better understanding of the relationship between aging and loss of *β*-cell mass.

#### 2.2.2. Islet Neogenesis

*β* cell proliferation is self-replication of the *β* cell, whereas islet neogenesis is the differentiation of progenitor cell or transdifferentiation of pancreatic non-*β* cells to *β* cells. Neogenesis of islets occurring during normal embryonic development and in very early postnatal life can lead to *β*-cell mass expansion. In addition to fetal [67] and neonatal [68] periods, *β* cell neogenesis has been shown to be important in increasing *β*-cell mass in the adult during periods of increased insulin demand such as obesity [42] and pregnancy [69]. Rosenberg et al. developed a model for islet neogenesis in adult mammalian pancreas in the 1980s, which showed that pancreatic ductal cells can be induced to differentiate into normal functioning adult endocrine cells [70]. *β*-cell neogenesis may occur through two pathways, stem/progenitor cell activation and transdifferentiation of adult pancreatic cells [71]. Islet neogenesis-associated protein (INGAP), found in pancreatic exocrine secretions, appears to stimulate the growth and proliferation of duct cells and their differentiation into endocrine cells [72–74]. INGAP and a bioactive 15 amino acid synthetic peptide (INGAP peptide) are inducers of islet neogenesis in a human islet system [73]. Other stimuli have been demonstrated to exert neogenic effects on the endocrine pancreas. The combination of epidermal growth factor (EGF) and gastrin has been shown to stimulate islet neogenesis in both animal and human studies [75]. Glucagon-like peptide 1 (GLP-1), an incretin from enteroendocrine L cells of the intestine, has been shown to induce islet neogenesis in rodents [76]. Although the potential for *β*-cell replication appears to decline substantially with age as evidenced by decreased PDX expression, the rate of islet neogenesis (expressed as percentage of insulin positive duct cells) is not affected by aging in humans [44].

In summary, various aspects of *β*-cell mass and function decline as a feature of age, thus contributing to the age-associated defects in insulin secretion. This defect when superimposed for an increased need for insulin, could contribute to impaired glucose homeostasis, glucose intolerance and diabetes. In the subsequent section, we will discuss and review literature on age-associated deterioration of insulin action.

## 3. Aging and Insulin Action

Insulin action is the ability of insulin to bind to its receptors located on tissues including muscle, liver, and adipose tissue and initiate signaling effects. The net physiological metabolic effects that result from insulin signaling include (a) regulation of glucose homeostasis through a decrease in hepatic glucose output (via decreased gluconeogenesis and glycogenolysis) and increase in glucose uptake, primarily into striated muscle and adipose tissue as well as (b) increase in lipid synthesis in fat cells, and attenuation of release of free fatty acid from triglycerides in fat. Insulin resistance results when normal circulating concentrations of the hormone are insufficient to regulate these processes appropriately.

### 3.1. Visceral Fat

It is well documented that aging is associated with a decline of insulin action [77–79]. The decline in insulin action with age is thought to contribute to the high prevalence of impaired glucose tolerance and Type 2 diabetes among the elderly [80, 81]. Notably, some studies support the hypothesis that the decline in insulin action in the elderly persons is related to increased abdominal fat rather than to aging per se [82]. However, other studies suggested that age-associated insulin resistance may not be explained solely by concomitant abdominal obesity [83]. Abdominal fat (also called visceral fat, VF), which is located in and around the viscera, has been demonstrated to be strongly related to many health conditions, including CVD, insulin resistance, and T2D [84]. Several cross-sectional studies suggest that visceral fat increases throughout the lifespan in men and women of all ages and race, independent of increases in body weight [85–88]. Many factors contribute to the increased VF seen with aging such as physiologic decline in GH/IGF-1 axis, decrease in sex steroids as well as sedentary life style [89]. The mechanisms how VF links to the metabolic syndrome are still not entirely clear, but it has been suggested to involve its anatomical location, leading to a “portal” effect of greater free fatty acids (FFAs) and glycerol release [90]. Gabriely et al. showed that extraction of VF from 20-month-old Fischer 344 Brown Norway (FBN) rats was sufficient to restore peripheral and hepatic insulin action to the levels of young rats. Moreover, removal of VF in Zucker Diabetic Fatty (ZDF) rats prevented the progressive decrease in insulin action and delayed the onset of diabetes, but VF extraction did not alter plasma free fatty acid levels [91]. Borst et al. reported that VF removal in Sprague Dawley (SD) rats tended to improve glucose tolerance and lowered some pro-inflammatory adipokines in serum; these animals displayed increased insulin-stimulated glucose transport in excised soleus and digitorum longus muscles as compared to control group. These studies provide verification that VF is a potent modulator of both hepatic and peripheral insulin action [92]. Calorie restriction (CR) extends life span and retards age-related chronic diseases in a variety of species, including rats, mice, fish, flies, worms, and yeast [93]. Our studies have shown that a reduction in fat mass, specifically VF, may be one of the possible underlying mechanisms of the antiaging effect of caloric restriction [94].

**Figure 3:**
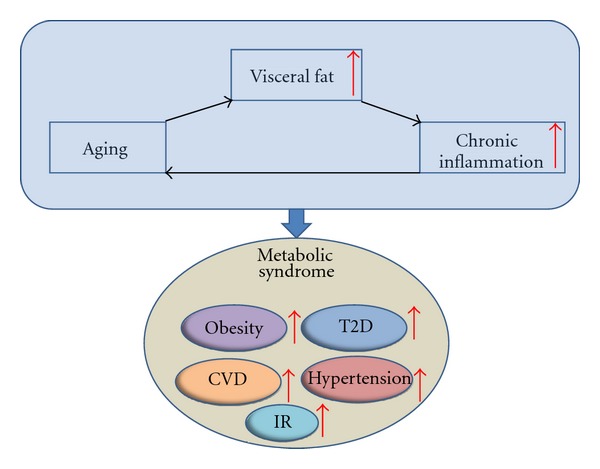
Link between Aging, visceral fat, inflammation, and metabolic syndrome. Visceral fat increases with age and the increase of visceral fat induces inflammation. Inflammation accelerates the process of aging. The vicious cycle of aging, visceral fat and inflammation increases the risk of metabolic diseases such as obesity, IR, T2D, CVD, and hypertension, CVD: cardiovascular disease, IR: insulin resistance, T2D: type 2 diabetes.

Nowadays, adipose tissue is recognized as an active metabolic-endocrine organ, and obesity is considered as a low-grade inflammatory condition strongly linked to adverse metabolic outcomes. A putative key link between increasing fat mass and obesity-related complications, including insulin resistance, is a chronic low-grade inflammatory state within adipose tissue, related to infiltration by macrophages [95]. We and others have shown that VF depots display a unique profile of inflammatory mediators compared to subcutaneous adipose tissue, including the clearly higher expression levels of macrophage migration inhibitory factor (MIF) and chemokine receptor 2 in VF [96, 97]. Other studies also suggested that VF is a stronger risk factor for metabolic disorders and mortality than subcutaneous fat [82, 98]. MIF, a proinflammatory cytokine, can activate nuclear factor (NF)-*κ*B signaling but directly inhibits the function of p53 [99, 100]. MIF knockout mice live longer than the control mice [101]. The possible explanation is aging is associated with inflammation and inflammation could accelerate aging process, whereas lack of MIF could downregulate NF-*κ*B, mediated inflammatory signaling, which will subsequently mitigate the process of aging [102]. Macrophages are considered to be a significant source for many fat-derived proinflammatory cytokines, and the percentage of macrophages in fat has been shown to increase in obesity [103]. Interestingly, our study suggested that the percentage of macrophages in the stromal vascular cell fraction from both visceral and subcutaneous fat increased with age regardless of obesity status [104]. Taken together, increase of VF is a hallmark of aging and a source of increased chronic inflammation. Inflammation could accelerate the aging process [105] and eventually lead to the metabolic dysfunction. Breaking this vicious cycle by decreasing the VF will be a potential therapeutic method for treating metabolic and related diseases (Figure 3).

### 3.2. Lipids and Insulin Resistance

Lack of or resistance to insulin leads to two metabolic crises: a marked increase in the rate of lipolysis in adipose tissue and activation of hepatic gluconeogenesis in spite of high plasma glucose levels. The increased rate of lipolysis increases circulating FFA levels, which, in turn, exacerbates insulin resistance in the whole body. It has been very well documented that the acute elevation of plasma FFA produces insulin resistance in both diabetic and nondiabetic individuals [106–108]. It is also shown that chronically elevated plasma FFA levels cause insulin resistance, and lowering elevated plasma FFA levels overnight normalizes insulin sensitivity in obese nondiabetic subjects and significantly improves insulin sensitivity in obese diabetic patients [109]. The mechanisms by which elevated levels of FFA produce insulin resistance have not been fully understood. However, studies have shown that increasing plasma FFAs acutely decreases insulin-stimulated glucose uptake and glycogen synthesis in human [110]. It is also reported that increase of FFA level in human inhibits PI3 kinase activity in skeletal muscle [111], suggesting the impairment of insulin signaling by FFA. Itani et al. pointed out that, with the infusion of FFA, increased accumulation of diacylglycerol (DAG) and protein kinase C (PKC) activity in muscle contribute to the impairment of insulin signaling [112], potentially through activation of NF-*κ*B [112]. The steatotic liver is also resistant to insulin in terms of inhibition of hepatic glucose production and stimulation of glycogen synthesis. The high FFA levels may be the unifying mechanism that accounts for the insulin resistance in obesity, type 2 diabetes, lipodystrophy, and aging [113]. We and others have shown that the circulating FFA levels are significant higher in 9- to 20-month-old SD rats compared to 3-month old, demonstrating that circulating FFA increases with age [113].

### 3.3. Aging and Central Insulin Resistance

Although peripheral insulin resistance is a hallmark of the development of T2D, more recent evidence has shown that insulin resistance also exists in central nervous system (CNS), and that central insulin action plays an important role in regulating whole body glucose metabolism. Like peripheral tissues, molecules in insulin signaling such as insulin receptor (IR), insulin receptor substrates (IRS), and phosphatidylinositol 3-kinase (PI3K) are universally expressed in the brain, indicating a potential role of insulin signaling in the brain. Koch et al. showed that mice lacking IR in CNS exhibit significantly more severe impairment of peripheral glucose homeostasis than mice lacking IR in the peripheral tissues [114]. Gelling et al. has shown that central infusion of PI3K inhibitor attenuated insulin-induced glucose lowering by 35%–40% in both acute and chronic insulin treatment paradigms, while hypothalamic overexpression of either IRS-2, a upstream kinase of PI3K, or protein kinase B (PKB/Akt), a key downstream mediator of PI3K action, enhanced the glycemic response to insulin by 2 folds in STZ-induced diabetic rats, suggesting that hypothalamic insulin signaling is an important determinant of the response to insulin in the management of uncontrolled diabetes [115]. Interestingly, an increasing body of evidence shows a link between diabetes and AD, a neurodegenerative disorder and the most common form of dementia. It has been reported that patients with T2D increase the prevalence of AD by two-to-three folds [116], and insulin levels and insulin activity in the central nervous system are reduced in AD [117]. Studies in human subjects show that both peripheral and central administration of insulin improves memory in AD patients [118–121], suggesting impairment of insulin signaling in the brain as a risk factor of neurodegenerative disorders, and restoration of insulin signaling could be a potential therapy for AD. This brings an interesting question whether aging is also associated with central insulin resistance. Fernandes et al. demonstrated that the protein levels of elements in the insulin signaling pathway such as IRs and SRC homology adaptor protein (SHC) did not change significantly in the forebrain cortex and cerebellum of rats aged 1 d to 60 wk. However, insulin induced tyrosine phosphorylation of IR and SHC, and the association of SHC/growth factor receptor binding protein-2 (GRB2) decreased significantly in both types of tissues [122]. Other studies showed that intracerebroventricular administration of insulin was more efficient at reducing food intake and body weight in 3-month-old rats than in 8- and 24-month-old rats, indicating the development of hypothalamic insulin resistance with age in Wistar rats. Furthermore, the tyrosine phosphorylation of IR and IRS-2 and the phosphorylation of downstream target genes such as Foxo1 and p70S6K declined, whereas serine phosphorylation of IR and IRS-2 increased with age in rat hypothalamus [123].

Aging-associated increase in central and peripheral insulin resistance could contribute to both diabetes and AD. The field of central insulin resistance and its role in the development of neurodegenerative disorders and the control of whole body glucose homeostasis is complicated and further studies are needed to fully understand the underlying mechanisms. For a detailed review of the insulin signaling in the brain, we refer the readers to the following reviews [9, 124–126].

## 4. Aging and Whole Body Glucose Homeostasis

It is clearly established that the risk for impaired glucose tolerance and diabetes increase with age in rodents and humans. The specific factors such as increased VF and circulating FFA that contribute to impaired insulin action and various defects in *β*-cell mass/function have been highlighted in the previous section. However, the integrated whole body glucose homeostasis is complex with various age-related parameters playing a crucial role on both aspects of glucose homeostasis, namely, insulin action and insulin secretion. Leptin, a hormone secreted from adipose tissue, plays a key role in energy intake and expenditure. Deficiency of leptin and its receptor leads to severe obesity, insulin resistance, and diabetes in rodents and humans. Resistant to the effects of leptin, termed leptin resistance, is seen in obesity and aging. Leptin resistance associated with aging [127–129] and decline in growth hormone (GH)/insulin-like growth factor (IGF)-1 axis [89] could play a key role in the alterations of glucose homeostasis in aging. Accumulating evidence suggests that endoplasmic reticulum (ER) stress plays a role in the pathogenesis of diabetes, contributing to both pancreatic *β*-cell function and peripheral insulin resistance [130]. It has been reported that aging is related to increase in proapoptotic markers with ER stress in multiple tissues, including lung, liver, kidney, and brain [131, 132]. In the past decade, a family of nicotinamide adenine dinucleotide- (NAD-) dependent protein deacetylases, termed sirtuins, have been shown to contribute to longevity. Sirtuins slow aging in worms, fruit flies, and mice [133]. Interestingly, overexpression of sirtuins or treat with activators of sirtuins, such as resveratrol protect against metabolic decline in aging, increases insulin sensitivity, increases insulin secretion, improves life quality, and extends lifespan [11, 117, 133]. In addition to the above-mentioned variables, aging-associated sedentary life style and diminished physical activity may be important factors for age-related changes of glucose homeostasis. Research has shown that healthy elderly with greater degrees of physical fitness have better glucose tolerance and lower level of insulin resistance than less active old people [134]. In addition, aging is associated with defects in the balance of insulin secretion and insulin action (demands). In the young, a hyperbolic relationship exists between insulin secretion and insulin sensitivity, whereby pancreatic *β* cell compensates for changes in whole-body insulin sensitivity through a proportionate increase in insulin secretion [116]. Our data shows that compared to younger animals, when challenged with a prolonged hyperglycemic stimuli older animals are unable to maintain the insulin secretion proportional to the degree of resistance [115].

Whole body glucose homeostasis is a complex balance of glucose production and utilization by different tissues. Food intake and hepatic glucose production are the two sources of glucose production, while skeletal muscle contributes to the majority of the glucose uptake and utilization. Utilizing tracer technology, it is possible to differentiate between the effects on glucose production (liver) and glucose utilization (primarily the muscle). Hepatic glucose production (HGP) plays crucial roles in glucose homeostasis, both in the fasting and postprandial states. In contrast to rodents, where there is an increase in HGP in age, there are no differences in either the basal hepatic glucose production or the dose-response curve of its suppression by insulin between young and old individuals [135]. The European Group for the Study of Insulin Resistance reported that hepatic glucose production does not increase with age, when adjusted for lean body mass [136]. Furthermore, hepatic glucose output has not been shown to be increased in elderly patients with T2D [137]. Thus, hepatic insulin resistance does not seem play a significant role in decreased glucose tolerance of elderly people [138].

As mentioned earlier, skeletal muscle is the major source of glucose utilization. Glucose is transported into the cells by glucose transporters. Through anaerobic and aerobic pathways, glucose is broken down to generate energy. GLUT4 is the major glucose transporter in skeletal muscle responsible for insulin-stimulated glucose uptake. Muscle GLUT4 protein level is not altered in obesity and T2D, however, its expression levels decline with age, and are related to insulin sensitivity in normal controls [139]. European Group for the Study of Insulin Resistance demonstrated that glucose uptake is not altered as a function of aging per se but is secondary to increased body fat accumulation [140]. Moreover, the decrease of lean body mass [141] and contractile strength with age are other factors that contribute to the reduction in insulin stimulated glucose uptake. The above factors, along with changes in body composition, accumulation of VF, and increase in circulating FFA levels, contributes to the decreased glucose uptake with age.

## 5. Conclusion and Perspective

Glucose intolerance, insulin resistance, and T2D associated with aging are leading causes of morbidity and mortality through its multiple complications as well as increases in the risk for multiple other age-related diseases such as cancer, stroke, cardiovascular diseases, Parkinson's disease, and AD [6]. Though various factors that contribute to the changes in glucose homeostasis are relatively well characterized, there are still areas that are not yet fully elucidated such as the roles of aging on *β*-cell mass and function, and the crosstalk between central and peripheral insulin action. A comprehensive understanding of all the defects that impair glucose homeostasis in the elderly will lead to development of appropriate, novel treatments that will substantially improve quality of life and over all life span.

## Abbreviations

AD: Alzheimer's disease
Cdk: Cyclin-dependent kinase
CNS: Central nervous system
CR: Calorie restriction
CVD: Cardiovascular disease
DAG: Diacylglycerol
ER: Endoplasmic reticulum
FFA: Free fatty acid
GCK: Glucokinase
GH: Growth hormone
GLP: Glucagon-like peptide
GLUT: Glucose transporter
GPDH: Glycerophosphate dehydrogenase
GRB2: Growth factor receptor binding protein-2
GSIS: Glucose stimulated insulin secretion
HGP: Hepatic Glucose Production
IAPP: Islet amyloid polypeptide
hIAPP: Human IAPP
IGF: Insulin-like growth factor
INGAP: Islet neogenesis-associated protein
IR: Insulin receptor
IRS: Insulin receptor substrate
K_ATP_: ATP-sensitive K^+^
LDCV: large dense core vesicles
MIF: Macrophage migration inhibitory factor
NAD: Nicotinamide adenine dinucleotide
NAFLD: Nonalcoholic fatty liver disease
NF: Nuclear factor
PI3K: Phosphatidylinositol 3-kinase
PKB/Akt: Protein kinase B
PKC: Protein kinase C
SD: Sprague Dawley
SHC: SRC homology adaptor protein
SNAP-25: Synaptosomal-associated protein of 25 kDa
SNARE: Soluble N-ethylmaleimide sensitive factor attachment protein receptor
STZ: Streptozotocin
T2D: Type 2 diabetes
TCA: Tri-carboxylic acid
VAMP: Vesicle-associated membrane protein
VF: Visceral fat
ZDF: Zucker Diabetic Fatty.
