# Corrigendum to “Pancreatic Function, Type 2 Diabetes, and Metabolism in Aging”

**DOI:** 10.1155/2017/2131060

**Published:** 2017-01-12

**Authors:** Zhenwei Gong, Radhika H. Muzumdar

**Affiliations:** ^1^Department of Pediatrics, Divisions of Endocrinology and Geriatrics, Children's Hospital at Montefiore, Albert Einstein College of Medicine, Bronx, NY 10461, USA; ^2^Department of Medicine, Divisions of Endocrinology and Geriatrics, Children's Hospital at Montefiore, Albert Einstein College of Medicine, 1300 Morris Park Avenue, Bronx, NY 10461, USA

In the article titled “Pancreatic Function, Type 2 Diabetes, and Metabolism in Aging” [1], there were missing attributions and errors in citations, which should be corrected as follows:

(1) In Section  2.1.3, Aging and Calcium and Potassium Channels, wording from Ammon et al. should have been quoted as follows: “Studies from Ammon and colleagues have shown that raising the glucose concentration from 3 to 5.6 and 16.7 mM had no effect on ^86^Rb^+^ efflux [a model for K^+^ efflux] from islets of 24-month-old male rats whereas that from 24-month-old female rats was decreased. … net uptake of ^45^Ca^2+^ was significantly diminished in islets of 24-month-old rats compared to islets of 3-month-old rats. … In the presence of 16.7 mM-glucose, islets of 24-month-old rats exhibited only 60–70% of the insulin release obtained with islets from 3-month-old rats. … These data suggest that the decreased insulin secretory response to glucose during old age is due, at least in part, to inadequate inhibition of K^+^ efflux and diminished net uptake of Ca^2+^” [29].

(2) In Section  2.1.4, Effects of Age on Insulin Granule Exocytosis, the wording “Calcium constitutes … protein targets” was taken from Lang et al. without quotation and should be replaced with the following: Calcium is the major stimulus as well as regulator of exocytosis of insulin [30–32].

(3) In Section  2.2.1, Apoptosis and Proliferation, the wording “*β*-cell mass … by 7 months of age” was taken from Rankin et al. without quotation or citation in that paragraph and should be replaced with the following: From newborn period to adulthood, there is an increase in mass and functional capacity of *β* cells to secrete insulin [51]. This increase in *β* cell mass also has been shown in adult rodents in pregnancy, a physiological state of increased insulin requirements [52]. *β* cell mass expansion in adult mice has been attributed to *β* cell neogenesis from pancreatic ducts or hematopoietic tissues [53] and differentiation of stem cells and specialized *β* cell progenitors as well as self-renewal by the *β* cell [54, 55, 57].

(4) In Section  2.2.1, Apoptosis and Proliferation, wording from Kushner et al. should have been quoted as follows: Kushner et al. report that “Prenatal islet development occurred normally in cyclin D2^−/−^ or cyclin D1^+/−^ D2^−/−^ mice. However, *β* cell proliferation, adult mass, and glucose tolerance were decreased in adult cyclin D2^−/−^ mice, causing glucose intolerance that progressed to diabetes by 12 months of age. Although cyclin D1^+/−^ mice never developed diabetes, life-threatening diabetes developed in 3-month-old cyclin D1^−/+^ D2^−/−^ mice as *β* cell mass decreased after birth. Thus, cyclins D2 and D1 were essential for *β* cell expansion in adult mice” [66].

(5) In Section  2.2.2, Islet Neogenesis, the wording “Islet neogenesis-associated protein … endocrine cells” was taken from Pittenger et al. without quotation and should be replaced with the following: Islet neogenesis-associated protein (INGAP) and a synthetic derivative of that protein, named INGAP peptide, have been shown to stimulate the growth and differentiation of duct cells into endocrine cells in dogs, hamsters, or human islets in vitro [72–74].
